# Toward Water and Oil Repellent Coating: Synthesis of Fluorinated Methacrylate-Glycidyl Methacrylate Copolymers

**DOI:** 10.1021/acsomega.4c03275

**Published:** 2024-08-02

**Authors:** Kubra Ozkan Hukum, Tugba Demir Caliskan, Tuncer Caykara, Gokhan Demirel

**Affiliations:** † Bio-inspired Materials Research Laboratory (BIMREL), Department of Chemistry, Faculty of Science, Gazi University, Ankara 06500, Türkiye; ‡ Department of Chemical Engineering, Faculty of Engineering, Ankara University, Ankara 06100, Türkiye

## Abstract

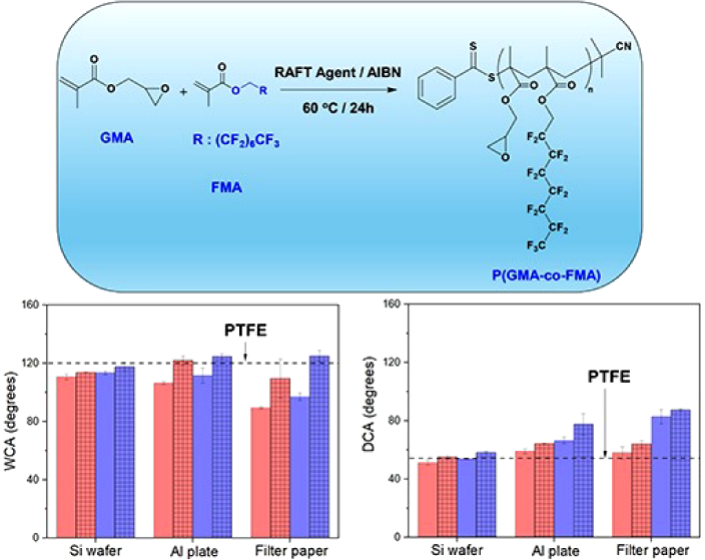

The development of durable and eco-friendly coatings with excellent adhesion and remarkable surface properties remains a critical pursuit in various industries. This study introduces an innovative methodology for the synthesis of glycidyl methacrylate-*co*-fluorinated methacrylate (P(GMA-*co*-FMA)) random copolymers with variable fluorine contents derived from GMA and FMA monomers. The copolymerization of these constituents yields coatings with enhanced durability and unique surface characteristics. Particularly, the incorporation of FMA introduces novel surface functionalities, leading to high water and oil repellent properties. The copolymer-coated surfaces exhibited impressive water contact angles ranging from 105° to 125° and decane contact angles ranging from 50° to 85°. The wettability of the P(GMA-*co*-FMA) coatings demonstrated a strong dependence on the fluorine content in the copolymers, with higher fluorine content resulting in superior water and oil repellency. Through a comprehensive characterization, we demonstrate the exceptional adhesion and self-cleaning capabilities of the fabricated films. Notably, the self-cleaning efficacy of P(GMA-*co*-FMA)-coated surfaces persists even following a prolonged duration of 6 months. Furthermore, our investigation reveals the influence of copolymer composition on surface wettability and contact angle hysteresis, providing valuable insights for tailoring coating properties. Overall, the novelty of this study lies in the synthesis of P(GMA-*co*-FMA) copolymer coatings with superior adhesion and self-cleaning properties. These advancements present promising applications in various fields, including electronics, textiles, and medical supplies, where such durable and functional coatings can significantly enhance product performance and longevity.

## 1 Introduction

Fouling, especially hydrocarbon-based fouling, poses a significant challenge with broad implications for industrial applications such as textiles,^1,2^ electronics,^3,4^ and oil/water separations.^5,6^ The adhesion of oils and organic substances to surfaces poses a substantial risk, potentially causing coating failures and adverse effects on various materials. An effective strategy to address fouling is surface modification using low-surface energy materials, which exhibit both hydrophobic and oleophobic properties. In addition, the incorporation of low-surface energy characteristics into the coatings not only enhances resistance to water-based contaminants but also provides robust defense against oils and other organic substances.

The application of low-surface energy materials holds great promise for developing durable coatings with potential applications in outdoor clothes,^7,8^ marine,^9,10^ and medical industries^11,12^ due to their self-cleaning,^13−15^ antifouling,^16−18^ and anticorrosion^19,20^ features. This multifaceted approach addresses fouling challenges across diverse industrial settings, offering a versatile and effective solution. Among low-surface energy materials, fluorinated-based materials (e.g., perfluoro silanes,^21,22^ perfluoro acids,^23,24^ and perfluoro polymers^25−27^) are particularly favored for their enhanced oil-repelling characteristics due to their lower surface energy compared to hydrocarbons (CH > CH_2_ > CH_3_ > CF_2_ > CF_2_H > CF_3_).^28−30^ Hare et al. conducted the synthesis of films by depositing perfluorododecanoic acid (PFDoA) onto metal surfaces. The surface energy of PFDoA films was determined to be 6 mN/m^2^,^29^ the lowest among known surface energies. However, contemporary environmental regulations have now imposed restrictions on the use of long-chain fluorocarbons (above C_7_).^31^ This is due to the potential toxicity and bioaccumulation of degradation products, such as perfluorooctanoic acid (PFOA)^32^ and perfluorooctane-sulfonate (PFOS),^33^ upon their eventual breakdown.^34,35^ Consequently, researchers focus on the utilization of shorter-chained fluorocarbons (C_7_ and below), which exhibit a reduced propensity for bioaccumulation.

To date, various fluorinated polymers (fluorinated poly(methacrylates/acrylates),^36,37^ fluorinated polyurethanes,^38,39^ and perfluoro-polyethers^21,27,40^ have been used as low-surface energy coatings. Moreover, for industrial applications, the stability of hydrophobic/oleophobic coatings is paramount. Consequently, the covalent attachment of coatings to the substrate surface becomes imperative to enhance their stability. It is known that many fluorinated polymers are unable to chemically attach to surfaces; hence, there is a growing interest in their copolymers.^41,42^ Glycidyl methacrylate (GMA) is a promising material for synthesizing durable hydrophobic/oleophobic coatings, acting as an anchor due to an active epoxy group capable of reacting with various functional groups (−OH, −COOH, and −NH_2_).^43−47^ Zou and colleagues employed the atom transfer radical polymerization (ATRP) method to synthesize poly(glycidyl methacrylate) (PGMA)-*block*-poly(2,2,2-trifluoroethyl methacrylate) (PTFEMA) copolymers, and then they were deposited onto cotton fabrics.^48^ The modified cotton fabrics exhibited outstanding water repellency, as evidenced by water contact angles (WCAs) of ∼163°. Notably, these superhydrophobic cotton fabrics displayed long-term stability, ultrahigh durability, and robustness.^48^ Several research obtained similar results when they used copolymers composed of PGMA and fluorinated polymers for grafting different surfaces.^49,50^ Mao and Gleason synthesized water and oil repellent coatings consisting of GMA and fluorinated methacrylate (FMA) with different −CF_2_ content, or fluorinated acrylate (FA) monomers, using the initiated-chemical vapor deposition (iCVD) method.^51^ They reported that the hardness and modulus of P(GMA-*co*-FMA) and P(GMA-*co*-FA) films were increased with increasing GMA content in the copolymers, whereas their liquid repellency enhanced with increased fluorine content in the copolymers.^51^ Klimov et al. also developed self-cleaning coatings based on diblock copolymers of PGMA and PFMA with fluorine atoms in the monomer unit ranging from three to seven. They also found that by increasing the fluorine content in the copolymers, water repellency was enhanced.^52^

To this end, P(GMA-*co*-FMA) copolymers have the capability to serve as water and oil repellent materials. To our knowledge, only a few articles have reported on grafting P(GMA-*co*-FMA) random copolymers onto surfaces to create hydrophobic/oleophobic nanocoatings.^51^ However, the stability of such coatings has not been documented in the literature. To fill this gap, we developed nanocoatings by grafting P(GMA-*co*-FMA) random copolymers. Specifically, P(GMA-*co*-FMA) copolymers were synthesized via RAFT polymerization and subsequently grafted onto substrates, including silicon wafers, aluminum surfaces, and filter paper. The coated substrates were then stored for 6 months under ambient conditions. The morphology of the coatings was analyzed using atomic force microscopy (AFM), and the surface wettability of the coatings was determined through contact angle measurements.

## 2 Experimental Section

### 2.1 Materials

Methacryloyl chloride (MACl, 97%), 2,2,3,3,4,4,5,5,6,6,7,7,8,8,8-pentadecafluoro-1-octanol (PF_oct_–OH, 98%), glycidyl methacrylate (GMA, ≥ 97%) monomer, triethyl amine (Et_3_N, ≥ 99%), and RAFT agent 2-cyano-2-propyl benzoithioate (CTA, >97%) were acquired from Sigma-Aldrich, while azobis(isobutyronitrile) (AIBN) initiator was purchased from Across Organic. Liquid monomers were passed through aluminum oxide before use to remove inhibitors. Aluminum oxide was purchased from Across Organics. Solvents such as dichloromethane (DCM, ≥ 99.8%), tetrahydrofuran (THF, ≥ 99.9%), ethyl acetate (EtAc), hexane (≥97%), decane(≥99%), and magnesium sulfate (≥99.5%) were also purchased from Sigma-Aldrich.

### 2.2 Synthesis of the FMA Monomer

**Figure 1:**
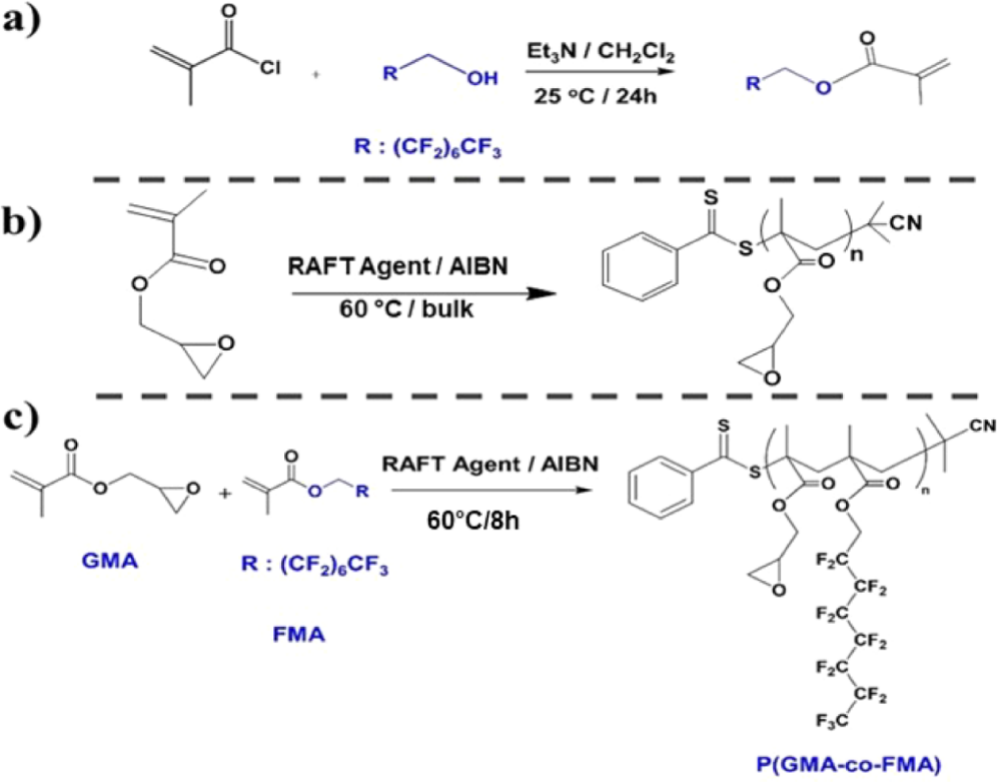
Synthesis scheme of the FMA monomer (a), PGMA homopolymer (b), and P(GMA-*co*-FMA) copolymer (c).

The fluorinated methacrylate (FMA) monomer was synthesized via a Schotten-Baumann reaction^50^ of methacryloyl chloride with perfluoro alcohol, detailed elsewhere^51^ (Figure 1a). Briefly, a 0.5 mL solution of MACl (1.010 mmol) in DCM was added dropwise to a solution of PF–OH (0.425 mmol) and Et_3_N (1.010 mmol) in dry DCM, which was stirred at 0 °C for 10 min. The final solution was stirred at room temperature for 24 h to allow the esterification reaction to proceed. Subsequently, Et_3_N:HCl salt was filtered off, and the crude product was extracted with DCM; the organic phase was washed with water and NaOH. Pure PFMA was obtained after purification by silica gel-packed column chromatography using an ethyl acetate/hexane solvent mixture (3:1, v:v).

### 2.3 Synthesis of the PGMA Homopolymer

Poly(glycidyl methacrylate) (PGMA) homopolymer was synthesized in bulk using the reversible addition–fragmentation chain transfer (RAFT) polymerization technique. In a typical RAFT polymerization, GMA monomer (34.3 mmol), RAFT agent (0.528 mmol), and AIBN (0.176 mmol) were used. The solution was charged in an ampule and degassed, and the ampule was sealed and placed in a water bath. The RAFT polymerization proceeded at 60 °C for 24 h. The crude product was precipitated in methanol and then dried under vacuum to yield PGMA polymer (Figure 1b).^53^

### 2.4 Synthesis of the P(GMA-*co*-FMA) Random Copolymer

The synthesis procedure of the P(GMA-*co*-FMA) random copolymer was reported elsewhere^54^ (Figure 1c). In this study, two P(GMA-*co*-FMA) random copolymers, named P(2) and P(25), were synthesized using GMA and FMA monomers, an AIBN initiator, and a RAFT agent. For the synthesis of the P(25) copolymer, the molar ratio of reactants was [FMA]_o_: [GMA]_o_: [AIBN]_o_: [CTA]_o_ = 1:1:0.007:0.02. Specifically, GMA (6 mmol), FMA (6 mmol) monomers, AIBN initiator (0.04 mmol), and RAFT agent (0.12 mmol) were mixed in a round flask and stirred under N_2_. RAFT polymerization proceeded at 60 °C for 8h. The crude product was first dissolved in THF and then precipitated in hexane. This process was repeated three times to obtain the pure P(GMA-*co*-FMA) random copolymer. For the synthesis of the P(2) copolymer, the similar procedure was applied with a modified molar ratio of [FMA]_o_: [GMA]_o_: [AIBN]_o_: [CTA]_o_ = 1:3:0.007:0.02.

### 2.5 Polymer Film Preparation

P(GMA-*co*-FMA) films were deposited on various substrates, such as Si wafers, aluminum plates, and filter papers through the dip coating method using 1.0 wt % polymer solution in THF. Before polymer depositions, all surfaces were rinsed with acetone and alcohol to remove dirt and dried. Subsequently, they were treated with oxygen plasma for 15 min to prefunctionalize surfaces for further grafting. Then, all surfaces were coated with P(GMA-*co*-FMA) polymers and annealed at 140 °C overnight. Unbounded polymers from films were also removed via rinsing surfaces with THF four times.

### 2.6 Characterization Techniques

Structural elucidation of the FMA monomer was carried out using grazing angle (GA) mode with an FTIR spectrometer (Thermo Nicolet 6700 GA-FTIR, WI USA). FTIR spectra were acquired in the scan range of 4000–400 cm^–1^, with a resolution of 4 cm^–1^. The results for each sample are presented as an average of 64 scans. In addition, nuclear magnetic resonance (^1^H and ^19^F NMR) spectroscopy (Bruker 400) was performed to determine the chemical structure of the FMA monomer and P(GMA-*co*-FMA) copolymer. All products were dissolved in CDCl_3_, and their NMR spectra were recorded at 400 MHz. In addition, RAFT polymerization was carried out at 60 °C for 8 h with the initial molar ratios, [FMA]_o_: [GMA]_o_: [AIBN]_o_: [CTA]_o_ = 1:1:0.007:0.02 to determine the polymerization rate of the P(GMA-*co*-FMA) copolymer. Samples from the polymerization solution were taken at different time intervals (2, 4, 6, and 8 h), and their Mn was calculated using NMR results.

Atomic force microscopy (AFM) was conducted with A Dimension 3100 microscope (Digital Instruments, Inc.) to image film morphologies. A 5 μm × 5 μm area of the films was scanned in tapping mode, and their root-mean-square (RMS) roughness was evaluated based on the recorded AFM image. The wettability of P(GMA-*co*-FMA) films was determined by contact angle measurements of deionized water (2 μL, 18 MΩ cm resistivity) and *n*-decane (2 μL) using a drop shape analysis software (DSA100, Krüss, Germany). *n*-Decane was used as a nonpolar liquid since contact angle measurements provide direct confirmation of the presence of fluorocarbon groups on surfaces. The thickness of P(GMA-*co*-FMA) films was obtained using an ellipsometer (model DRE, EL X20C) with a He–Ne laser (λ = 632.8 nm) at an incident angle of 75°. The advancing and receding contact angles of water on the films were determined by inflating or deflating the drop via an automatic syringe. Additionally, the durability of the films was evaluated using a tape peel-off test, where adhesive tape (3 M Scotch tape) was applied to the film surface and then peeled off at a 90° angle. This procedure was repeated 10 times, and following each cycle, the contact angle of water on the films was measured.

## 3 Results and Discussion

### 3.1 Synthesis of the FMA Monomer

A series of experiments were conducted to synthesize the fluorinated methacrylate monomer and then poly(glycidyl methacrylate-*co*-perfluoro methacrylate) (P(GMA-*co*-FMA)) random copolymers utilizing the RAFT polymerization method. Initially, the fluorinated methacrylate was synthesized via a polycondensation reaction of MACl with PF_oct_–OH. The selection of this short-chain perfluoro alcohol, PF_oct_–OH, was deliberate, as it exhibits nontoxic attributes in comparison to its longer perfluoro alcohol counterparts.^31^ This strategic approach in experimental design and synthesis not only enhances the precision of the polymerization process but also ensures the incorporation of fluorinated components with desirable properties in the resultant polymers.

**Figure 2:**
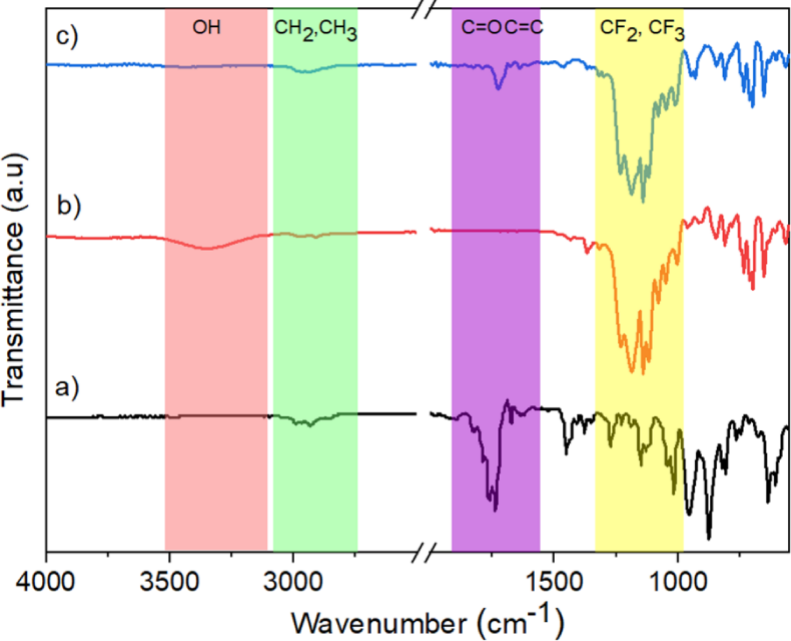
GA-FTIR spectra of (a) MACl, (b) PF_oct_–OH, and (c) FMA monomers.

The chemical structure of the FMA monomer was elucidated using GA-FTIR, with additional analysis through online spectral databases for organic compounds.^55^ The IR spectra, depicted in Figure 2, confirmed the successful synthesis of FMA via the employed synthetic procedure. Notably, the IR spectra of FMA exhibited distinctive peaks corresponding to ester stretching (−O–C=O) and −C–O–C stretching at approximately 1730 and 1269 cm^–1^, respectively. These features emanate from the acid chloride reaction with alcohol during the synthetic process. Furthermore, the −OH stretching vibration in the range of 3500–3400 cm^–1^ vanished, while the −CF_2_ and −CF_3_ stretching vibrations in the region of 1200–1100 cm^–1^ emerged. The presence of −C=C– stretching vibration at 1639 cm^–1^ was also identified. As a result, the FMA monomer was successfully synthesized, rendering it conducive for subsequent polymerization processes.

**Figure 3:**
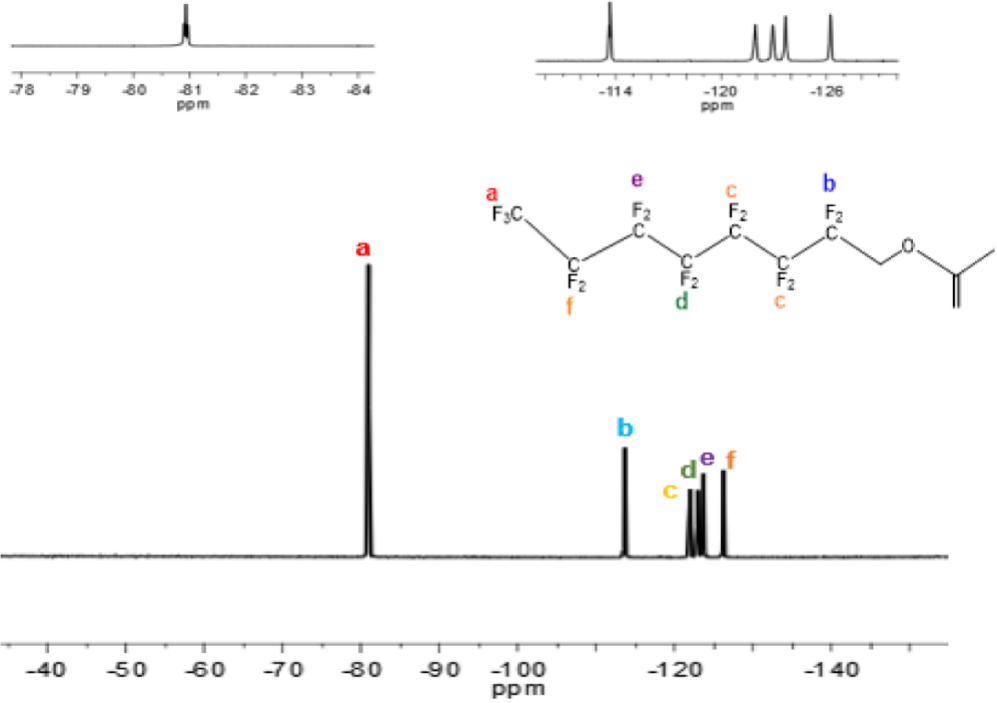
^19^F-NMR spectra of the FMA monomer.

To further elucidate the chemical structure of the FMA monomer, a comprehensive^19^F-NMR analysis was conducted, with the corresponding spectra illustrated in Figure 3. The distinctive features in the spectra include a triplet peak at 81 ppm (a), corresponding to the fluorine atoms in −CF_3_ groups. Multiple peaks at −113.5 ppm (b) are attributed to the CF_2_ group, proximal to the CH_2_ group (−CF_2_–CH_2_CH_2_–O−). Additionally, a singlet peak at −122 ppm (c) was associated with −CF_2_– groups bonded to the −CF_2_–CH_2_ group (−CF_2_–CF_2_–CH_2_CH_2_–O−). Other singlet peaks at −123 ppm (d), −124 ppm (e), and −126 ppm (f) are attributed to the −CF_2_– groups in proximity to the −CF_2_–CF_2_–CH_2_– group, −CF_3_–CF_2_- group, and −CF_3_ group, respectively. In conclusion, NMR data also conclusively validate the successful synthesis of FMA.

### 3.2 Synthesis and Characterization of the PGMA Polymer

**Figure 4:**
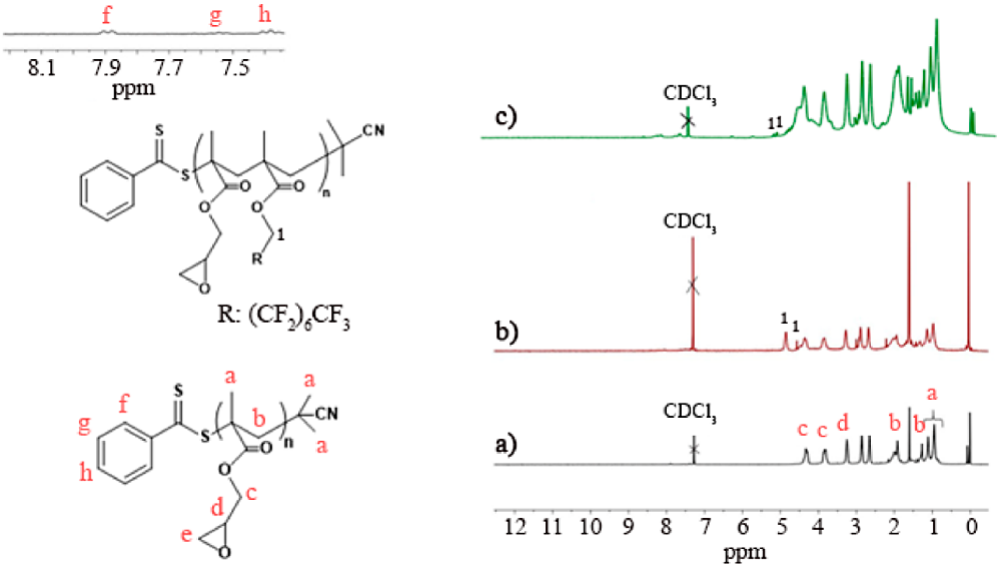
^1^H NMR spectra of (a) PGMA, (b) P(25) copolymer, and (c) P(2) copolymer.

The PGMA homopolymer was synthesized using the RAFT polymerization technique. Specifically, GMA (34.3 mmol), CTA (0.528 mmol), and AIBN (0.176 mmol) were mixed without using any solvent. The polymerization was carried out at 60 °C for 24 h under N_2_ gas. The PGMA polymer was precipitated in methanol. The chemical structure of the synthesized PGMA homopolymer was characterized by ^1^H NMR spectroscopy (Figure 4a). Upon examination of the ^1^H NMR spectrum, the multiple peaks at 3.35–3.10 ppm (d) and double quartet peaks at 2.65–2.85 ppm (e) were attributed to the −CH proton and −CH_2_ protons in the epoxy group of GMA, respectively. In addition, the C=C bond of the GMA monomer converted to a C–C bond during the polymerization. Thus, the double peaks at around 6.15–5.35 ppm, attributed to the C=C bond disappeared, and new double peaks at 1.4–1.8 ppm (b) belonging to the C–C bonds were formed. As a result, PGMA polymer was successfully synthesized. The molecular weight of the synthesized PGMA shown in Table 1 was calculated theoretically and experimentally using ^1^H NMR (please see Supporting Information S1 for detailed calculation).

**Table 1:** Molecular Weight of PGMA and P(GMA-co-FMA) Copolymers and their Film Thickness on Si Wafers

	Mn (^1^H NMR) (g/mol)	Mn (theoretical) (g/mol)	thickness of films on Si wafers (nm)
PGMA	10430	6320	43.28 ± 13.6
P(2)	19562	17255	51.44 ± 10.9
P(25)	25889	20314	65.15 ± 9.2

### 3.3 Synthesis of the P(GMA-*co*-FMA) Copolymer

The copolymerization of P(GMA-*co*-FMA) was conducted with different ratios of GMA to FMA monomers, specifically 1:1 and 3:1. Structural elucidation was achieved through NMR analysis, and the overall NMR data unequivocally confirmed the successful synthesis. Figure 4b,**c** illustrates the ^1^H NMR spectra of the obtained P(GMA-*co*-FMA) copolymer after an 8-h reaction, with the PGMA polymer serving as a reference. Notably, all spectra corresponding to the PGMA polymer were present in the NMR spectra of the P(GMA-*co*-FMA) copolymer. The multiple peaks at 2.75–2.95 ppm (e) attributed to the −CH_2_ protons for epoxy groups of GMA, and the double quartet peaks at 7.3–7.9 ppm (f,g,h) belonged to −CH protons and −CH_2_ protons of the benzene ring in RAFT agent part. Furthermore, distinctive multiple peaks at 4.5–4.75(*) ppm were observed, corresponding to the CH_2_ groups adjacent to the CF_2_ group of FMA. These findings provide a detailed insight into the structure of the synthesized P(GMA-*co*-FMA) copolymer. Based on the NMR results, the composition of FMA in P(GMA-*co*-FMA) copolymers was found to be 25% and 2% when FMA:GMA ratio used in polymerization was 1:1 and 1:3, respectively. Herein, copolymers based on FMA content were named as P(2) and P(25). In addition, Table 1 shows the molecular weight of P(2) and P(25) copolymers that was calculated theoretically and experimentally using ^1^H NMR (please see Supporting Information S2 for detailed calculation).

**Figure 5:**
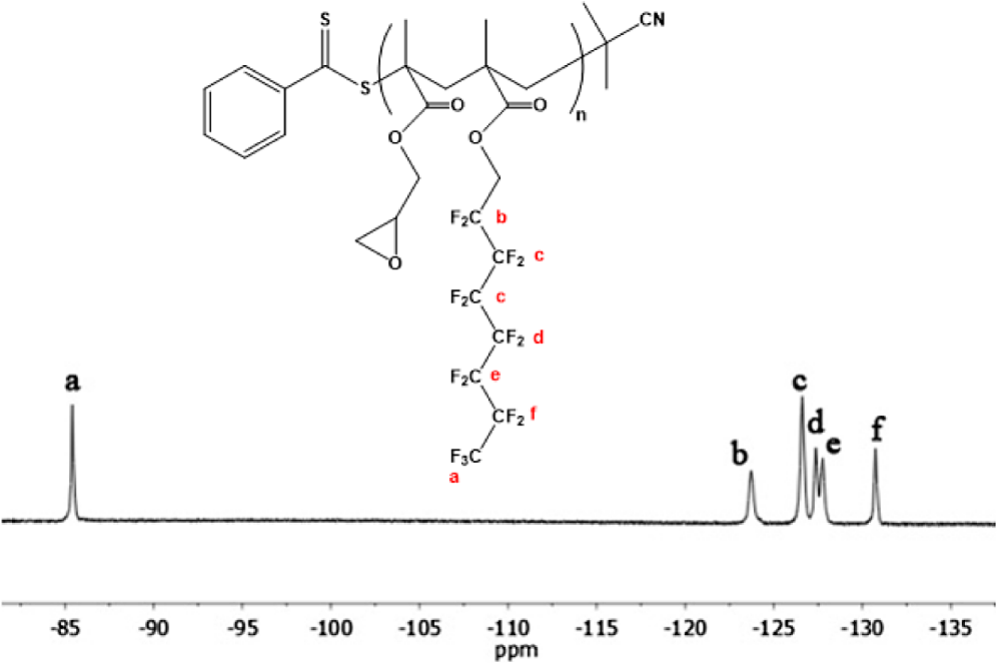
^19^F NMR spectrum of the P(GMA-*co*-FMA) copolymer.

The ^19^F NMR spectrum of the P(GMA-*co*-FMA) copolymer is also presented in Figure 5. In the spectrum obtained in CDCl_3_ for P(GMA-*co*-FMA), the triplet at −85 ppm corresponds to fluorine in the CF_3_ group (a) adjacent to −CF_2_, the multiplet at −123 ppm corresponds to fluorine in CF_2_ group (b) adjacent to −CH_2_, the singlet at around −126 ppm corresponds to fluorine in the CF_2_ group (c) adjacent to the −CF_2_CH_2_–, and the singlets at −127.5 ppm (d), −128 ppm (e), and −131 ppm (f) correspond to fluorine in the CF_2_ group adjacent to −CF_2_CF_2_–, −CF_3_CF_2_–, and CF_3_ (f), respectively (Figure 5). According to the NMR results, the P(GMA-*co*-FMA) copolymer was successfully synthesized. No side products were detected within the copolymer. Consequently, a pure P(GMA-*co*-FMA) copolymer was obtained.

### 3.4 Kinetic Features of the Polymerization of the P(GMA-*co*-FMA) Copolymer

Experimental criteria such as the linear change in molecular weight with monomer conversion, a constant concentration of active centers, and narrow molecular weight distribution, collectively indicate the occurrence of a living free radical polymerization.^56^ To characterize the living nature of P(GMA-*co*-FMA) RAFT polymerization, experiments were conducted at 70 °C for different time intervals (2, 4, 6, and 8 h) with the initial molar ratios [FMA]_o_: [GMA]_o_: [AIBN]_o_: [CTA]_o_ = 1:1:0.07:0.02. The molecular weights of PGMA and P(GMA-*co*-FMA) copolymers synthesized over the 8-h polymerization period were determined theoretically and experimentally through NMR analysis (Supporting Information S2). According to the results, the Mn of PGMA was determined to be 10723 g/mol using NMR data, whereas the theoretical Mn for the PGMA polymer was calculated to be 8962 g/mol. Table 2 presents the conversion rates and molecular weights of P(GMA-*co*-FMA) copolymers. Notably, the polymerization process conducted for 8 h yielded a high conversion rate of 94%. Furthermore, it was observed that the maximum Mn for the copolymers exceeded 20k indicating successful synthesis of high molecular weight copolymers within the specified reaction time. These findings highlight the efficacy of the polymerization process in achieving desired molecular weights and conversion rates.

**Table 2:** Monomer Conversion and Molecular Weight of P(GMA-*co*-FMA) Copolymers

polymerization duration (hours)	conversion (%)	Mn (^1^H NMR) (g/mol)	Mn (theoretical) (g/mol)
2	27	10430	6320
4	63	16182	13658
6	81	19520	17492
8	94	25889	20314

**Figure 6:**
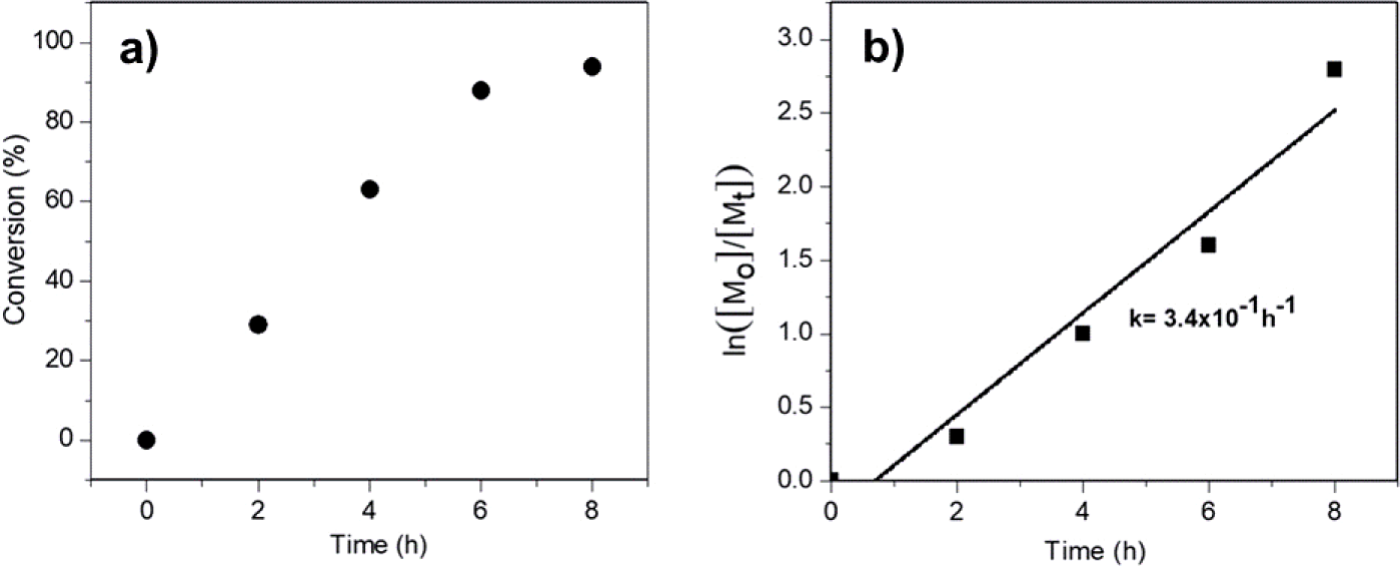
a) Changes in monomer conversion with polymerization time. (b) Kinetics of solution-phase polymerization.

In addition, Figure 6 provides the kinetic study of RAFT polymerization in the solution phase. As depicted in Figure 6a, monomer conversion exhibited a linear increase with polymerization time, reaching up to 63% within the first 4 h. Subsequently, a deviation from linearity occurred, and the conversion plateaued at 94%. The ln([M_o_]/[M_t_]) as a function of time plot in Figure 6b clearly illustrates the pseudo-first-order kinetics of the RAFT polymerization. The observed rate constant for the reaction was determined to be 3.4 × 10^–1^ h^–1^ from the slope of the obtained straight line. The linear increase in molecular weight with increasing monomer conversion, the pseudo-first-order nature of the reaction degree, and its consistent progression all provide evidence of the living nature of the polymerization.^56^

### 3.5 Morphological Analysis of the P(GMA-*co*-FMA) Film Surface

A series of experiments were conducted to fabricate PGMA and P(GMA-*co*-FMA) films. Initially, silicon (Si) wafers were dip-coated into 1% solutions of PGMA and P(GMA-*co*-FMA) in chloroform, followed by drying at room temperature. The thicknesses of the resulting films were evaluated and demonstrated in Table 1. It is well-established that film thickness is influenced by the molecular weight of the polymers utilized. Accordingly, among the three polymers examined, PGMA exhibited the lowest Mn, while the P(25) copolymer possessed the highest Mn. Consequently, the thickness of PGMA films on Si wafers averaged around 43.28 ± 14 nm, whereas those of P(2) and P(25) copolymer films measured 51.44 ± 11 nm and 65.15 ± 9 nm, respectively. This variation in film thickness highlights the role of polymer molecular weight in dictating film morphology.

**Figure 7:**
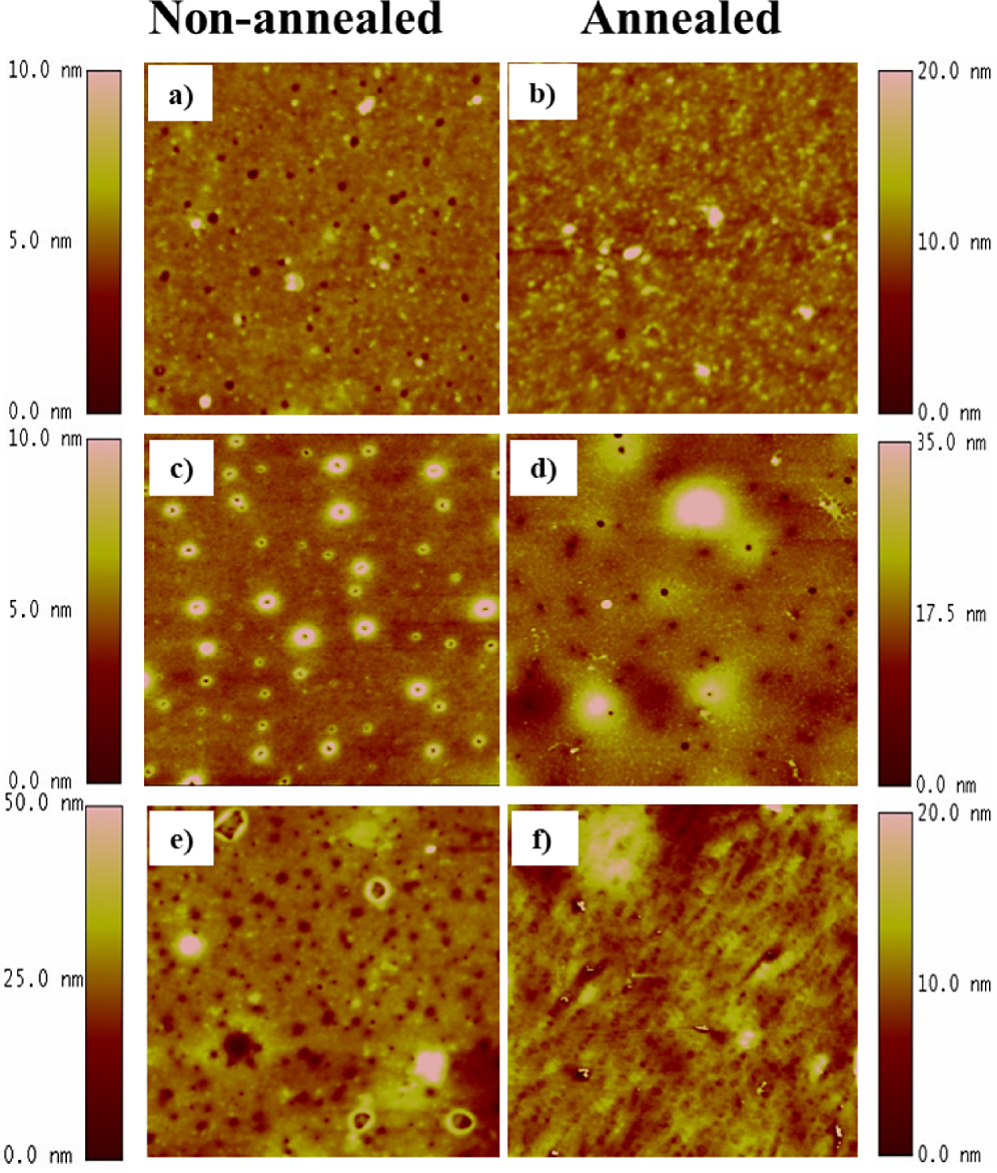
AFM (5.0 × 5.0 μm^2^) topographical images of PGMA and P(GMA-*co*-FMA) coating surfaces before and after annealing at 140 °C for 3 h. (a, b) PGMA coating (*RMS*_*a*_ ≈ 0.40 nm, *RMS*_*b*_ ≈ 0.96 nm); (c, d) P(2)-based coating *(RMS*_*c*_ ≈ 0.69 nm, *RMS*_*d*_ ≈ 2.85 nm), and (e, f) P(25)-based coatings (*RMS*_*e*_ ≈ 3.38 nm and *RMS*_*f*_ ≈ 4.51 nm).

Atomic force microscopy (AFM) analysis was performed to assess the surface morphology of P(GMA-*co*-FMA) films before and after annealing at 140 °C for 3 h. AFM images of PGMA films were also included for comparative analysis. As depicted in Figure 7, the PGMA anchoring layers, obtained through dip-coating, exhibited a smooth and homogeneous surface. However, upon copolymerization with the FMA monomer, a distinctive change in surface morphology occurred due to phase separation. The AFM images revealed the presence of circular domains attributed to FMA chains on the P(GMA-*co*-FMA) films. Both PGMA and copolymerized films exhibited smooth surfaces, characterized by low roughness values (<4 nm). Notably, the annealing process did not exert a significant influence on surface roughness, maintaining the film surfaces in a smooth state even after annealing.^23^

### 3.6 Wetting Features of P(GMA-*co*-FMA) Coatings

**Figure 8:**
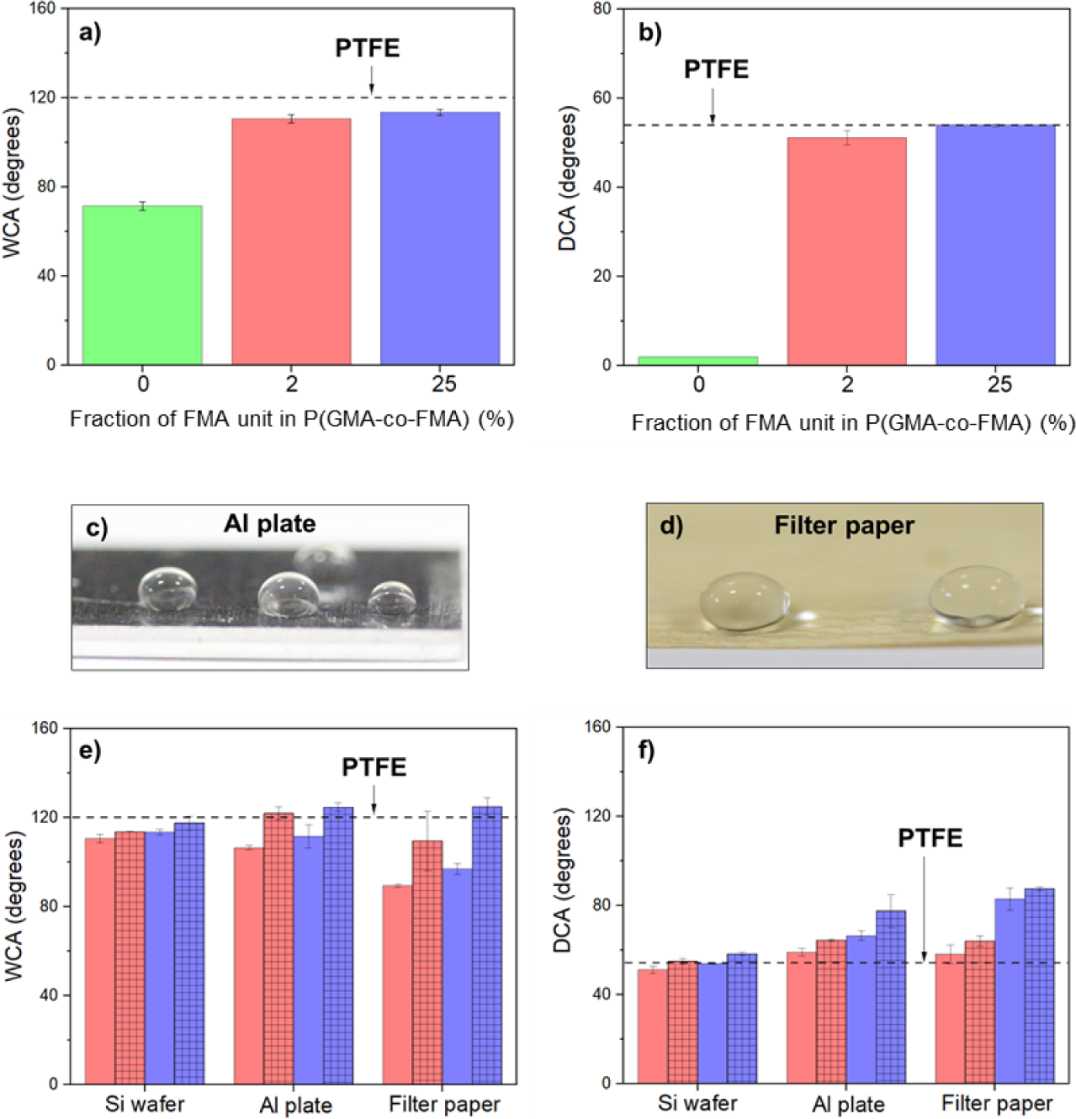
WCA (a) and DCA (b) for P(GMA-*co*-FMA) films of different FMA content on Si wafer surface before annealing (0% samples were coated with only PGMA). Water droplet on P(25)-coated Al plate (c) and P(25)-coated filter paper (d). WCA (e) and DCA (f) for P(GMA-*co*-FMA) films of different FMA content (red:2%, purple:25%) on Si wafer, Al plate, and Filter paper surfaces before annealing (solid) and after annealing (mesh). The contact angle for PTFE is also given for comparison.

The water and oil repellency of P(GMA-*co*-FMA) and PGMA coatings were assessed through static contact angle (CA) measurements of water (WCA) and decane (DCA). Substrates, including Si wafers, Al plates, and filter papers, were initially coated with PGMA and P(GMA-*co*-FMA) copolymers containing 2% and 25% FMA units. Subsequently, 2.0 μL of liquids was applied to the surfaces, followed by the measurement of their contact angles. Notably, all substrates are inherently wettable with both water and decane, with filter papers exhibiting absorption characteristics due to their porous nature. Figure 8a illustrates that PGMA-coated Si wafers display partial wettability with water (WCA ≈ 71.4°) and complete wettability with decane (DCA < 5°). Similar wetting features were observed for PGMA-coated other substrates, as summarized in Table S1. However, the copolymerization of P(GMA-*co*-FMA) influences the water and oil repellency of PGMA films. The incorporation of only 2% FMA chains in the copolymer notably enhances the WCA and DCA of the films. For P(2)-coated Si wafers, increasing from 71.4° to 110.6° (Figure 8a), and the DCA rises to 51.2° (Figure 8b). The 25% FMA content in the copolymer maintains a significant increase in DCA (DCA ≈ 54.9°), while WCA shows a marginal change (WCA ≈ 113.7°). In comparison to the water and oil repellency of P(GMA-*co*-FMA)-coated films with the PTFE film, the latter demonstrates greater hydrophobicity. However, an increase in FMA content in copolymers imparts superior oleophobic features compared to PTFE. Consequently, it is deduced that the addition of FMA in the PGMA polymer enhances the water and oil repellency of flat surfaces.

It is well-known that surface roughness emerges as a key parameter influencing liquid repellency. Consequently, Al plates and filter paper, characterized by inherently rough surfaces, were subjected to the P(GMA-*co*-FMA) film coating. Contact angle measurements reveal a notable improvement in water and oil repellency for Al plates upon FMA incorporation into the copolymer (Figure 8c). Specifically, after the deposition of P(2) copolymer, Al plates exhibit complete hydrophobicity (WCA ≈ 106.5°) and partial oleophobicity (DCA ≈ 59.1°) (Figure 8e,f). Moreover, the subsequent increase in FMA content in copolymers induces a significant change in their WCA and DCA values (WCA ≈ 111.6° and DCA ≈ 66.3°). In contrast, filter paper exhibited a distinct behavior. Despite its prior tendency to absorb with PGMA coating, the wettability of filter paper was contingent upon the FMA content in the copolymer (Figure 8d–f). Films comprising 2% FMA conferred water repellency to the filter paper (WCA ≈ 89.5°), albeit lower than the same film-coated silicon (Si) wafer (WCA ≈ 110.6°). This was anticipated, as dispersion of water on a hydrophobic porous surface may occur, resulting in a decrease in the contact angle—a phenomenon known as the Wenzel state.^57,58^ Conversely, these films exhibited enhanced oil repellency (DCA ≈ 58.2°) compared to P(GMA-*co*-FMA)-coated Si surfaces (Figure 8e/f). Notably, a shift toward oleophobicity (DCA ≈ 83°) was achieved when the copolymer contains 25% FMA. These results showed the interplay between copolymer composition, surface roughness, and the resulting liquid repellency characteristics, with the potential for tailoring these properties based on specific applications.

The annealing process emerges as another critical factor for enhancing hydrophobicity and oleophobicity, particularly when polymeric films contain fluoro carbon groups (e.g., −CF_2_, −CF_3_) within their structure. During annealing, these fluorinated groups tend to migrate toward the surface owing to their lower surface energy. In our investigation, P(GMA-*co*-FMA) films with FMA compositions of 2% and 25% were subject to annealing at 140 °C overnight, and their corresponding water contact angles (WCA) and decane contact angles (DCA) are presented in Figure 8e,f, respectively. Remarkably, all annealed films demonstrated significantly elevated levels of water and decane repellency, highlighting the effectiveness of the annealing process in enhancing the hydrophobic and oleophobic properties of the films. Comparative analysis with PTFE film revealed that only annealed P(GMA-*co*-FMA)-coated Al and filter paper surfaces exhibited higher WCA values than those of PTFE. Moreover, all annealed surfaces, including flat ones, demonstrated superior oleophobicity compared to PTFE. This observation emphasizes the efficacy of the annealing process in enhancing both hydrophobic and oleophobic characteristics of P(GMA-*co*-FMA) films, leading them as promising materials for applications requiring advanced liquid repellency.

**Figure 9:**
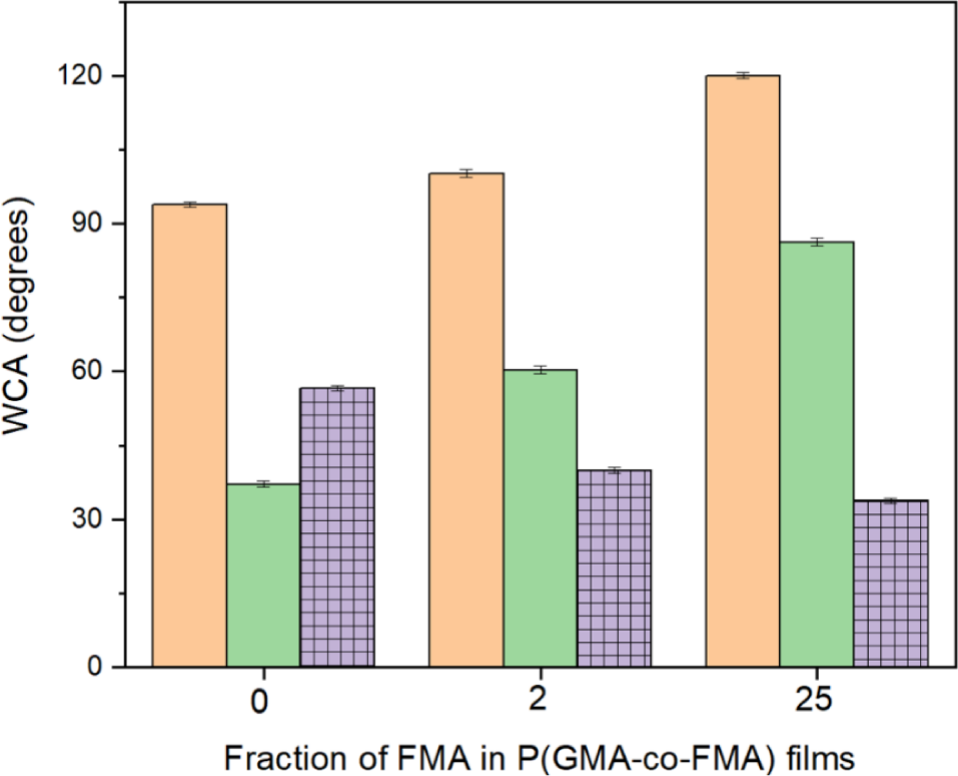
Changes in advancing (orange bar) and receding (green bar) angles of PGMA and P(GMA-*co*-FMA) films for water and their contact angle hysteresis (meshed purple bar).

Figure 9 presents the dynamic contact angles of water on PGMA and P(GMA-*co*-FMA) films. The difference between the advancing (θ_A_) and receding (θ_R_) contact angles is defined as contact angle hysteresis (*CA*_*H*_ = θ_A_ – θ_R_). A reduction in *CA*_*H*_ values facilitates easier droplet rolling-off, contributing to highly self-cleaning properties. As depicted in Figure 9, both advancing and receding angles on films increased with higher FMA content, while *CA*_*H*_ decreased. Notably, when comparing fluorinated films, slight variations in *CA*_*H*_ values were observed. In conclusion, the presence of FMA in the P(GMA-*co*-FMA) films led to a decrease in surface wettability, resulting in easier droplet rolling. This finding underscores the significance of copolymer composition in modulating the wetting behavior and self-cleaning properties of the films.

### 3.7 Durability and Stability of Hydrophobic and Oleophobic P(GMA-*co*-FMA) Films

The long-term durability of coatings is significantly influenced by their adhesion to substrates. When coatings physically adhere to substrates, they are susceptible to delamination during mechanical deformation processes, such as peeling, abrasion, washing, or storing, under ambient conditions. However, when coatings are chemically bonded to substrates, they exhibit enhanced durability, thereby retaining their physical and chemical surface properties, such as oil/water repellency, anticorrosion, and antifouling characteristics. The glycidyl methacrylate (GMA) monomer plays a crucial role in achieving highly durable coatings due to its epoxy groups, which have the ability to react with various functional groups, such as −OH, −COOH, and −NH_2_. Therefore, we synthesized P(GMA-*co*-FMA) copolymers via the copolymerization of GMA with FMA monomers. These copolymers were subsequently deposited onto oxygen plasma-treated Si wafers and Al plates, followed by annealing at 140 °C overnight. The resulting films exhibited remarkable water and oil repellency.

**Figure 10:**
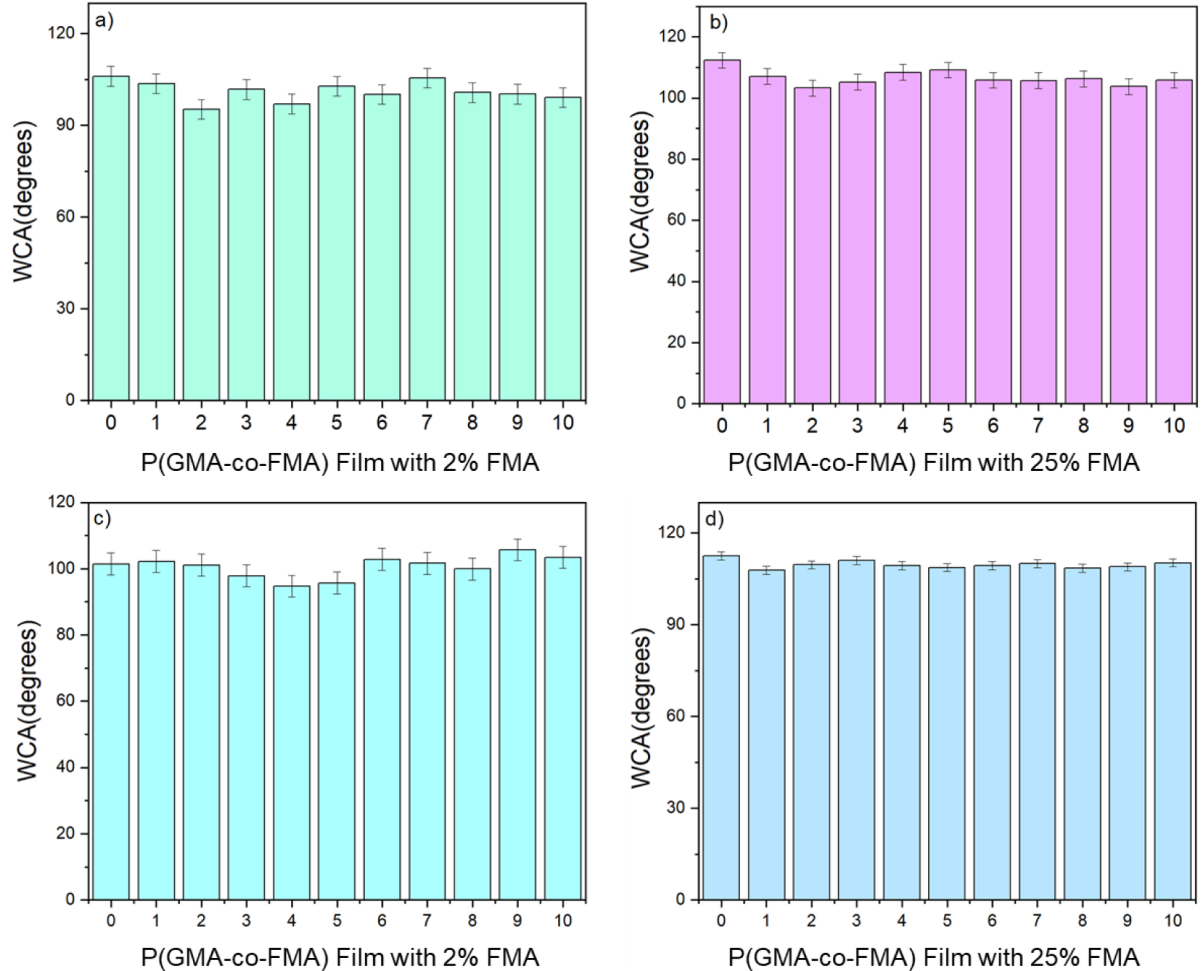
WCA on P(GMA-*co*-FMA) films deposited on Si wafers (a, b) and Al plates (c, d) after peel-off test for 10 cycles.

To evaluate the adhesion of P(GMA-*co*-FMA) films to both Si wafers and Al plates, a tape peel-off test was conducted. In this method, adhesive tape (3 M Scotch tape) was applied to the film surface and then peeled off at a 90° angle.^58^ This procedure was repeated 10 times, and following each cycle, the contact angle of water on the films was measured (Figure 10). Remarkably, it was observed that the water contact angle (WCA) of all films exhibited minimal change even after undergoing the peel-off test for 10 cycles. The films maintained their highly hydrophobic nature throughout the testing process, indicating robust adhesion to the substrates (Si wafers and Al plates). This substantiates the chemical adherence of the P(GMA-*co*-FMA) films to the substrate surfaces, rendering them resistant to removal. These characteristics underscore the potential utility of P(GMA-*co*-FMA) films as coatings in various applications, including automotive and electronics, where delamination is a critical concern.

**Figure 11:**
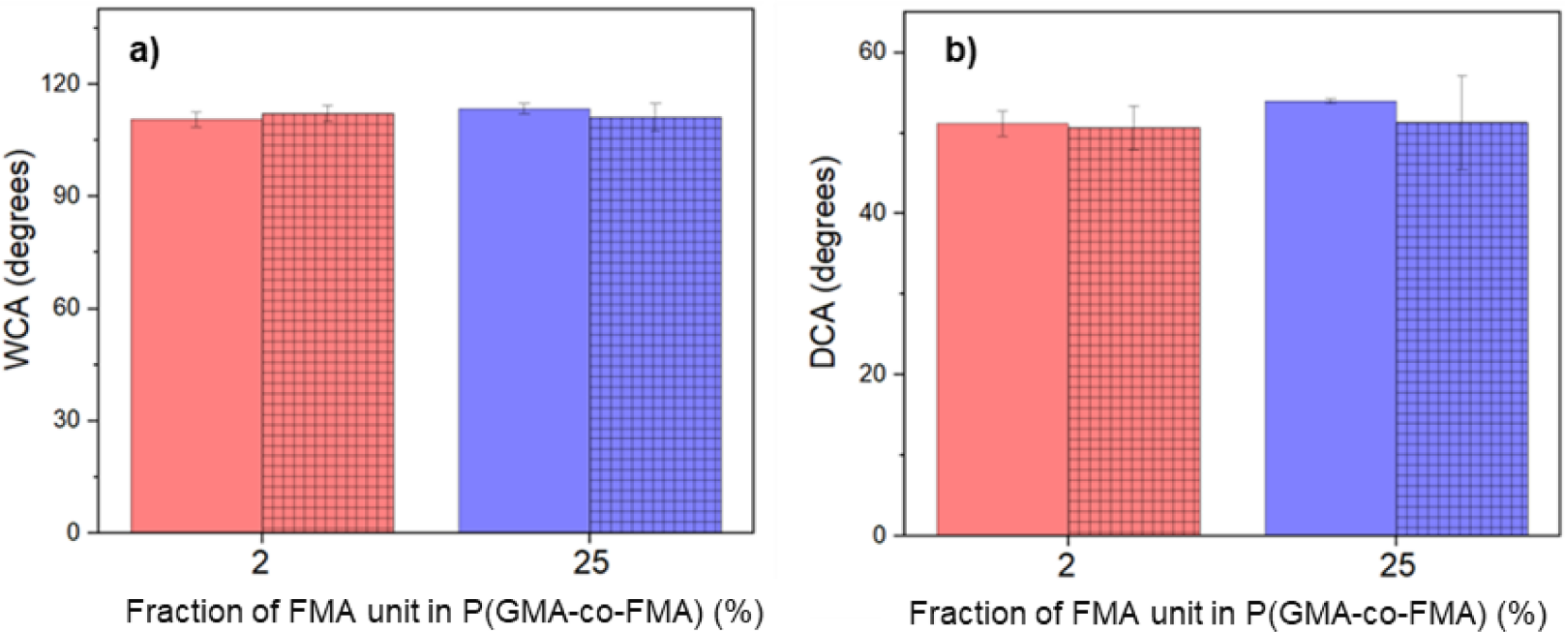
Wettability changes of P(GMA-*co*-FMA) films on Si wafer after storage for 6 months. (a) WCA and (b) DCA.

The evaluation of the stability of P(GMA-*co*-FMA)-coated Si wafers was also conducted through CA measurements of water and decane following a 6-months storage under ambient conditions. Figure 11a,b illustrates that the water and oil repellency of the films, irrespective of FMA content, did not undergo significant changes. This consistent performance underscores the robust nature of these coatings. Their enduring ability to resist water and oil, along with sustained antifouling properties, positions them as exceptionally promising materials for diverse applications in commercial and industrial protective coatings. The observed changes in repellency further emphasize the potential utility and durability of P(GMA-*co*-FMA) coatings, indicating their suitability for a range of protective coating applications.

### 3.8 Surface Energy Estimation and Stability of P(GMA-*co*-FMA) Coatings

**Figure 12:**
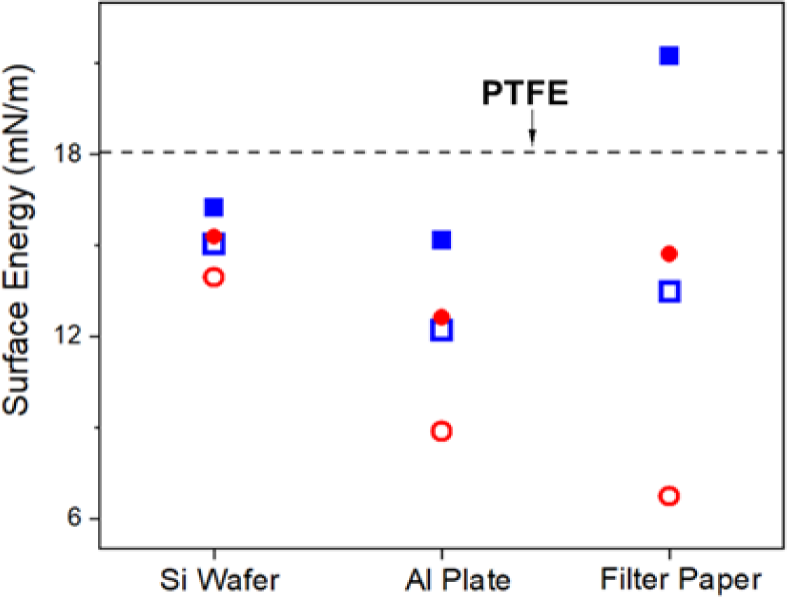
Surface energy of P(GMA-*co*-FMA) films. (Blue empty box) P(2)-based film and (solid red circle) P(25)-based film before annealing (solid blue box) and after the annealing (empty red circle) (a). The surface energy of PTFE film is also given for comparison. Wettability changes of P(GMA-*co*-FMA) films on Si wafer after storage for 6 months. (b) WCA and (c) DCA.

Surface energy (σ) provides valuable insights into the surface properties of P(GMA-*co*-FMA)-coated films. To estimate their surface energies, the Owens-Wendt method was applied utilizing water contact angle (WCA) and decane contact angle (DCA) results, as detailed in Supporting Information S4. The surface energy values of PGMA-coated substrates (Si wafer, Al plate, and filter paper) are tabulated in Table S3. Despite all surfaces being uniformly coated with PGMA, irrespective of their flat or rough topography, the surface energy of films remains consistent at approximately 36.4 ± 0.4 mN/m. Conversely, the deposition of P(GMA-*co*-FMA) significantly reduces the surface energy of film surfaces. As shown in Figure 12, at 2% FMA content, the surface energy of P(2)-based film is 16.3, 15.2, and 21.3 mN/m for Si wafer, Al plate, and filter paper, respectively. The relatively higher value for filter paper is attributed to the dispersion of water on the surface. With an increase in FMA content in the copolymer, the surface energy further decreases. It is noteworthy that annealing proves to be more effective in reducing the surface energy of films than increasing FMA content. This is anticipated as fluorinated carbon groups migrate to the surface during annealing, enriching the top surface layer and leading to a reduction in surface energy. Furthermore, it is observed that all annealed P(2)-coated surfaces exhibit lower surface energy than their nonannealed P(25) coated counterparts. Consequently, annealed P(25) surfaces emerge with the lowest surface energy among all films.

## 4 Conclusions

In conclusion, a series of P(GMA-*co*-FMA) random copolymers with varying FMA content were synthesized via the RAFT polymerization method. Notably, these copolymers are devoid of environmentally detrimental long-chain perfluoroalkyl segments. Substrates, including Si wafers, aluminum plates, and filter paper, were coated with these copolymers to prepare durable hydrophobic and oleophobic coatings. This study demonstrated that the incorporation of FMA monomer in copolymerization with GMA monomer resulted in the facile migration of FMA to the film surface, imparting substantial water and oil repellency to the thermoplastic boundary. The wettability of P(GMA-*co*-FMA) films was found to be contingent on both the FMA content in copolymers and the annealing process. Remarkably, even at a low concentration (2% FMA), copolymerization of GMA with FMA enabled the attainment of noteworthy levels of oil and water repellency. Furthermore, these films exhibited highly durable hydrophobicity and oleophobicity features even after the peel-off test for 10 cycles and maintained oil and water repellency effectiveness for more than 6 months. In conclusion, the absence of environmentally harmful perfluoroalkyl segments, coupled with the sustained hydrophobicity and oleophobicity under mechanical deformation and the long lifespan of these coatings, makes them feasible for various applications. They are suitable for use in medical supplies, the consumer electronics industry, and protective clothing for the military. Specifically, these coatings can be used to coat medical tubing, endoscope lenses, surgical visors, and shields. Additionally, oleophobic coatings have been extensively used to create smudge-resistant touchscreens for smartphones and tablets. Moreover, these coatings are employed in protective clothing against chemical warfare agents, highlighting their potential to enhance safety and functionality in diverse fields.

## Supporting Information Available

Calculation of molecular weight of the PGMA polymer (S1), calculation of molecular weight of P(GMA-*co*-FMA) copolymers (S2), wettability of PGMA-coated surfaces (S3) and estimation of surface energy for P(GMA-*co*-FMA) films (S4) (PDF)
