# Supplementary material for: Toward Water and Oil Repellent Coating: Synthesis of Fluorinated Methacrylate-Glycidyl Methacrylate Copolymers
Source: ACS Omega. 2024 Aug 2;9(32):34650–60. doi: 10.1021/acsomega.4c03275 (PMC11325509; doi:10.1021/acsomega.4c03275)
Supplement: Supplementary file 1 — ao4c03275_si_001.pdf [file ao4c03275_si_001.pdf]

SUPPORTING INFORMATION

Toward Water and Oil Repellent Coating: Synthesis of Fluorinated Methacrylate-Glycidyl Methacrylate Copolymer

Kubra Ozkan Hukum [1], Tugba Demir Caliskan[1,2], Tuncer Caykara[1], Gokhan Demirel[1*]*

[1] Bio-inspired Materials Research Laboratory (BIMREL), Department of Chemistry, Faculty of Science, Gazi University, Ankara 06500, Türkiye

[2] Department of Chemical Engineering, Faculty of Engineering, Ankara University, Ankara 06100, Türkiye;

Email: gdemirel@gazi.edu.tr, and tgbdemir@ankara.edu.tr

Supporting Information

S1. Calculation of molecular weight of PGMA homopolymer

The theoretical molecular weight of PGMA (Mn = 8962 g mol^{-1}) homopolymer was determined using the following Eqn 1.[2]

$$\overline{M}_{n,th} = \frac{[M]_0}{[CTA]_0} x\ a\ x\ M_{MW} + M_{CTA} \qquad (1)$$

where M_{MW} and M_{CTA} denote the molecular weights of the monomer and the RAFT agent, respectively, while $[M]_0$ and $[CTA]_0$ represent the initial concentrations of the monomer and the RAFT agent. Symbol a signifies monomer conversion.

Utilizing ^{1}H NMR measurements, the molecular weight of PGMA was also determined as 10723 g mol^{-1}, leveraging the integration areas of the –Ar-H peak at 7.9 ppm for the CTA molecule and the –CH_2 peaks in the range of 3.8-4.2 ppm for PGMA polymer.

S2. Calculation of molecular weight of P(GMA-co-FMA) copolymers

The theoretical molecular weights of P(GMA-co-FMA) copolymers were calculated using Eqn 2 as follows.

$$\overline{M}_{n,th} = \frac{[M]_0}{[CTA]_0} x\ a\ x\ M_{MW} + M_{CTA} \quad (2)$$

$$M_{MW} = x_{GMA}\, M_{GMA} + x_{FMA} M_{FMA} \quad (2a)$$

where x_{GMA} and x_{FMA} denote the mole fraction of GMA and FMA, respectively, while M_{GMA} and M_{FMA} represent the molecular weight of GMA and FMA monomers, respectively.

In addition, molecular weight of P(GMA-co-FMA) determination using ^{1}H-NMR spectroscopy involved acquiring spectra for each polymer sample. The integral areas of peaks corresponding to the C-H protons at δ 1.4 ppm from the methyl groups in the RAFT agent structure within the polymer chain, alongside the peak intensity of –CH_2 groups at δ 2 ppm in the P(GMA-co-FMA) copolymer, facilitated the calculation of polymer molecular weights at different polymerization stages.

S3. Wettability of PGMA-coated surfaces

Table S1. Surface wettability of PGMA-coated surfaces

	WCA (°)	DCA (°)
Si wafer	71.4 ± 1.97	wet
Aluminum plate	70.5 ± 1.84	wet
Filter paper	71.9 ± 3.01	wet

S4. Estimation of surface energy for P(GMA-co-FMA) films

The surface energy of PGMA, P(GMA-co-FMA) films were calculated according to Owens-Wendt method (Equation S2) (1).

$$\gamma_{l1}(1 + cos\theta_1) = 2\sqrt{\gamma_s^d \gamma_{l1}^d} + 2\sqrt{\gamma_s^p \gamma_{l1}^p}$$

$$\gamma_{l1}(1 + cos\theta_2) = 2\sqrt{\gamma_s^d \gamma_{l2}^d} + 2\sqrt{\gamma_s^p \gamma_{l2}^p}$$

$$\gamma_s = \gamma_s^d + \gamma_s^p$$

where γ_s and γ_l are the surface tensions of the solid and liquid, respectively. The subscripts d and p correspond to the dispersion and polar components of the surface tension, respectively. Surface free energy (γ_s) and it's polar (γ_s^p) and dispersion (γ_s^d) components of the P(GMA-co-FMA) surfaces were determined using two sets of contact angle measurements of water and decane. The γ_l^p and γ_l^d components of liquids shown in Table S2 were used in the calculations.

Table S2. The γ_l^p and γ_l^d components of liquids

	γ_l^p	γ_l^d	γ_l
decane	0	23.8	23.8
water	21.8	51	72.8

Table S3. Surface energy of PGMA-coated surfaces

	γ_s (mN/m)
Si wafer	36.30
Al plate	36.84
Filter paper	36.03

References

1. Owens, D. K.; Wendt, R. C., Estimation of the surface free energy of polymers. Journal of Applied Polymer Science 1969, 13 (8), 1741-1747

2. Izunobi, J.U. and Higginbotham, C.L., Polymer molecular weight analysis by ^{1}H-NMR spectroscopy. Journal of Chemical Education 2011, 88(2), 1098-1104.
